# Multi-Omics and Phenotypic Analysis Reveal *Paenibacillus polymyxa* JX-1 as a Broad-Spectrum Biocontrol Agent Against Clubroot Disease

**DOI:** 10.3390/microorganisms14030520

**Published:** 2026-02-24

**Authors:** Shu Che, Jiankun Hu, Jiaqin Fan, Liping Yang, Rong Huang

**Affiliations:** 1 Jiangxi Provincial Key Laboratory of Agricultural Non-Point Source Pollution Control and Waste Comprehensive Utilization, Institute of Plant Protection, Jiangxi Academy of Agricultural Science, Nanchang 330200, China; shuche0523@163.com (S.C.);; 2 Laboratory of Bacteriology, Department of Plant Pathology, Nanjing Agricultural University, Nanjing 210095, China; 3 Institute of Grain Production, Yunnan Academy of Agricultural Science, Kunming 650205, China

**Keywords:** *Paenibacillus polymyxa*, biocontrol, fungal, bacteria, clubroot

## Abstract

*Paenibacillus polymyxa* is a promising biocontrol agent based on many applications in agriculture. In this study, characterization of a novel strain JX-1 was performed by various comparative phenotypic assays including cell wall-degrading enzyme activity assay, antagonism in vitro and in planta and multi-omics analysis consisting of whole-genome sequencing and metabolomic analysis. The results showed that JX-1 produced protease, cellulase, and pectinase, with protease activity exceeding other *P. polymyxa* strains. The supernatant and VOCs produced by JX-1 both contain components that effectively antagonize both bacteria and fungi. JX-1 inhibited *Fusarium verticillioides*, *Sclerotinia sclerotiorum*, *Botrytis cinerea* and *Sclerotium rolfsii*. Furthermore, it significantly reduced the severity of clubroot in *Arabidopsis thaliana* and *Brassica rapa* in planta, resulting in an improvement in the total shoot fresh weight. The closely related *P. polymyxa* strain JX-2 (ANI = 99.96% compared to strain JX-1) was used for comparative phenotyping and multi-omics analysis that implicated extracellular proteases and some small peptides could be essential for clubroot biocontrol. Genomic analysis of strain JX-1 confirmed that it is indeed *P. polymyxa* (ANI > 95% compared to reference strains). Moreover, JX-1 harbors predicted carbohydrate-active enzymes (CAZymes), 16 extracellular protease genes, and 17 gene clusters with biosynthetic potential (NRPS/PKS hybrids). Metabolomic profiling of the culture supernatant further identified differential accumulation of hydrophobic-amino-acid-containing small peptides, providing a metabolic basis for the observed antagonism and offering leads for future mechanistic studies. These findings establish JX-1 as a broad-spectrum biocontrol agent against clubroot disease.

## 1. Introduction

The management of soil-borne diseases poses a major global challenge to agricultural sustainability and food security. Among these, clubroot disease, which is caused by the obligate biotrophic pathogen *Plasmodiophora brassicae*, represents a particularly persistent threat to cruciferous crop production worldwide [1,2,3]. The pathogen’s long-lived resting spores can persist in soil for decades, leading to severe yield losses and significant economic damage [2]. The difficulty in genetically manipulating *P. brassicae* has hindered in-depth research into its pathogenic mechanisms, complicating the development of effective control strategies [2,4].

Currently, few commercially registered biological agents are available for clubroot management [5,6,7]. Meanwhile, overreliance on chemical pesticides contributes to environmental pollution and public health concerns due to residue accumulation [8,9]. Therefore, the development of environmentally sustainable and effective control measures against this devastating disease is urgently required.

Biological control agents (BCAs), primarily composed of beneficial microorganisms, offer a promising eco-friendly approach [10,11,12] for suppressing plant diseases through multiple mechanisms including direct antagonism, induced plant systemic resistance, promotion of plant growth and competition for ecological niches [13,14,15,16,17].

Although biocontrol strains like those from *Trichoderma* and *Bacillus* have been applied against clubroot for decades [6,18,19], most studies have been limited to phenotypic experiments, with relatively few investigations into biocontrol mechanisms. Recently, with the development of high-throughput omics, research has been increasingly devoted to the study of biocontrol bacterial strains [20,21]. Within the repertoire of promising BCAs, the genus *Paenibacillus* has attracted considerable attention. Members are known to synthesize a diverse array of antimicrobial compounds, enhance plant immunity, and improve rhizosphere microbiome structure [6,22,23,24]. Particularly, *Paenibacillus polymyxa* is one of the most famous and useful species from the genus *Paenibacillus* [24,25,26].

However, despite its recognized potential, research on the application of *P. polymyxa* specifically for the biocontrol of cruciferous clubroot remains notably limited. Although the application of *P. polymyxa* for clubroot control has been previously documented, systematic studies evaluating its efficacy against a broader spectrum of soil-borne diseases, as well as investigations into its underlying mechanisms, remain limited.

To bridge these knowledge gaps, we isolated and characterized a novel strain of *P. polymyxa*, evaluating its broad-spectrum antagonistic activity against major soil-borne pathogens and its specific efficacy in suppressing *P. brassicae*. The novelty of this work lies in the identification of the endophytic strain JX-1, isolated from eggplant stems in Jiangxi Province, China, which exhibits not only significant clubroot control capability but also strong inhibition against multiple fungal and bacterial pathogens.

To systematically investigate the biocontrol mechanism of strain JX-1, which exhibits specific anti-clubroot effects, an integrative multi-omics approach was adopted. Comparative genomic analysis revealed functional genetic features, including specific biosynthetic gene clusters, while metabolomic profiling identified a diverse array of antimicrobial metabolites in its culture supernatant. By correlating genomic potential with metabolic responses, we demonstrated the strain’s multi-layered antagonistic strategy and propose that extracellular proteases and specific small peptides are likely key components of JX-1’s unique biocontrol mechanism against *P. brassicae*. These findings provide valuable insights and a potential microbial resource for developing efficient and sustainable strategies for clubroot disease management.

## 2. Materials and Methods

### 2.1. Strains and Culture Conditions

All strains were cultured in Lysogeny Broth (LB) medium, Luria Agar (LA) medium, Potato Dextrose Agar (PDA) medium, Yeast Peptone Dextrose Adenine (YPDA) Medium and Potato Dextrose Water medium under 220 rpm at 28 °C [27]. The JX-1 strain was isolated from the stems of eggplant plants infected by *Solanum melongena* showing mild symptoms in Jiangxi Province, China, in 2023. Through isolation and molecular identification, it was determined to be *Paenibacillus polymyxa*. The strain was designated ‘JX-1’ as it was first identified in Jiangxi Province. The strain was deposited in the China General Microbiological Culture Collection Center (CGMCC) with the accession number CGMCC No. 33781. The other *P. polymyxa* strain used for comparative analysis, designated JX-2, was isolated from the same host plant in the same region and the same plant stem. All strains used in this study are presented in Table 1.

### 2.2. Phylogenetic Tree Analysis of 16S rRNA

Genomic DNA of strain JX-1 was extracted using the Bacterial Genomic DNA Extraction Kit (TransGen, Beijing, China), following the manufacturer’s protocol. The nearly full-length 16S rRNA gene was amplified by polymerase chain reaction (PCR) using the universal primers 27F (5′-GAGAGTTTGATCCTGGCTCAG-3′) and 1492R (5′-ACGGATACCTTGTTACGACT-3′). The PCR mixture contained 2× TransStart^®^ FastPfu PCR SuperMix (TransGen, Beijing, China), template DNA, and primers, under the following thermal cycling conditions: initial denaturation at 95 °C for 2 min; 40 cycles of 95 °C for 20 s, 58 °C for 20 s, and 72 °C for 60 s; and a final extension at 72 °C for 5 min. The amplified product was purified and sequenced by Sangon Biotech (Shanghai, China). The obtained 16S rRNA gene sequence was blasted via National Center for Biotechnology Information (NCBI). The sequence of strain JX-1 and related reference sequences retrieved from GenBank were stored in the TXT document and aligned using MEGA 11 software. A neighbor-joining method was used with 1000 bootstrap replicates [28].

### 2.3. Transmission Electron Microscopy (TEM) of Paenibacillus polymyxa JX-1

A 20 μL aliquot of each resuspended sample was applied dropwise to 200-mesh copper grids and incubated at room temperature for 10 min. Subsequently, the grids were negatively stained with 2% phosphotungstic acid for 3 min, and excess liquid was blotted using filter paper. Samples were then observed under an HT7800 transmission electron microscope (HITACHI, Tokyo, Japan).

### 2.4. Screening of JX-1 Biocontrol Activity Against Clubroot Disease Caused by Plasmodiophora brassicae

The resting spores of *P. brassicae* were extracted as described previously [29]. *Arabidopsis thaliana* ecotype Col-0 and Chinese cabbage (*Brassica rapa* subsp. *pekinensis*) cultivar ‘Jingyan 4’ were used in this study. Seeds were surface-sterilized with 75% ethanol, rinsed thoroughly with sterile water, and sown on a mixture of peat-based compost and vermiculite. Plants were grown in a greenhouse under the following conditions: 12 h light/12 h dark cycle, 24 °C, and 75% relative humidity. The *P. brassicae* isolate used in this study was identified as the highly virulent pathotype 4 (Pb4), which has the broadest host range, based on the standard Williams system. Two-week-old *A. thaliana* seedlings and 3-day-old Chinese cabbage seedlings were used in this experiment. The biocontrol experiment was conducted in round plastic pots (10 cm in diameter) filled with a sterile peat-based substrate.

A single colony of *P. polymyxa* JX-1 was cultured in fresh YPDA medium at 28 °C with shaking (200 rpm) for 7 days to prepare the fermentation broth, which contained approximately 1 × 10^8^ colony-forming units (CFU) per mL. For each plant, 1 mL of this bacterial culture (or its cell-free supernatant) was applied to the soil around the root zone. Six hours after this treatment, 1 mL of *P. brassicae* resting spore suspension (10^7^ spores/mL) was inoculated in the same manner. The spore concentration was verified and adjusted using a hemocytometer under a light microscope, following purification via a 50% sucrose density gradient centrifugation method adapted from previous studies [21]. Plants were maintained under controlled environmental conditions as described above. Disease development was assessed one month after pathogen inoculation. Plants were carefully uprooted, gently washed, and photographed to record gall formation. The fresh weight of the aerial parts was measured for each plant. Statistical analysis was performed based on the fresh weight data of shoots. The assay included six biological replicates per treatment group.

### 2.5. Dual-Culture Plate Assay of JX-1 Biocontrol Activity Against Fungus

The method was previously described [30]. The antagonistic activity of JX-1 strains against fungus was assessed using a dual-culture plate assay. A mycelial spot of fungus was placed at the left of a PDA plate, and the JX-1 strain was inoculated at the right of the plate. The plates were then incubated at 28 °C for 7 days. The fungal colony diameter was measured and the inhibition rate was calculated. The inhibition rate (%) was determined using the following formula: inhibition rate (%) = [(colony diameter of control − colony diameter of treatment)/colony diameter of control] × 100%. Each assay was repeated with three to five replicates.

### 2.6. Volatile Organic Compound (VOC) Antifungal Activity Assay

VOC antifungal assays were performed as previously described [12]. The biocontrol strains and antagonistic pathogens were separately inoculated onto LB and PDA media. The lids of the Petri dishes were discarded, and the dishes were inverted and tightly sealed together using a parafilm. The sealed Petri dishes were arranged with the biocontrol strain at the bottom and the pathogen on top, and incubated at 28 °C for 7 days. Sterile water streaks were used as the control. The colony diameter of the fungal pathogens was observed and recorded daily. The inhibition rate (%) was determined using the following formula: inhibition rate (%) = [(colony diameter of control − colony diameter of treatment)/colony diameter of control] × 100%. Each assay was repeated with three to five replicates.

### 2.7. Cell-Free Supernatant Antifungal Activity Assay

JX-1 was inoculated into fresh YPDA medium and cultured at 28 °C with shaking at 220 rpm for 7 days. The culture was then centrifuged at 6000 rpm for 3 min to collect the supernatant. The supernatant was filtered through a 0.22 µm filter to ensure sterility. The cell-free supernatant of JX-1 was diluted 1:100 (*v*/*v*) with PDA medium to prepare assay plates. A 5 mm mycelial plug of the indicator fungus was placed at the center of each plate. The plates were incubated at 28 °C for 7 days, after which the presence of inhibition zones was observed and photographed. The inhibition rate (%) was determined using the following formula: inhibition rate (%) = [(colony diameter of control − colony diameter of treatment)/colony diameter of control] × 100%.

### 2.8. Cell and Cell-Free Supernatant Antibacterial Activity Assay

JX-1 was inoculated into fresh YPDA medium and cultured at 28 °C with shaking at 220 rpm for 7 days. The culture was then centrifuged at 6000 rpm for 3 min to collect the supernatant and cell pellet. Resuspend the cells in sterile water to an OD_600_ = 1.0. The supernatant was filtered through a 0.22 µm filter to ensure sterility. The concentration of the pathogenic bacteria was adjusted to an OD_600_ of 1.0 and evenly spread onto the surface of LA medium. We inoculated 2 microliters of the bacterial resuspension at the center of the medium. A hole was made in the center of the agar using a hole puncher, and 10 µL of the filtered supernatant was added into the hole.

The plates were incubated at 28 °C, and inhibition zones were observed after 36 h. Sterile medium with pathogenic bacteria was used as a negative control. A 2 µg/mL kanamycin (Km) solution was used as the positive control. The plates were incubated at 28 °C for 36 h, after which the presence of inhibition zones was observed and photographed.

### 2.9. Biofilm Formation Assay

Biofilm formation was quantified using the crystal violet staining method as described by O’Toole and Kolter (1998) [31]. In brief, each tested strain was first cultured overnight at 28 °C with shaking in fresh LB medium. The overnight culture was then diluted 1:100 in fresh LB medium and incubated under the same conditions until the optical density at 600 nm (OD_600_) reached 1.0. Subsequently, each bacterial suspension (4 mL) was incubated statically in sterile glass tubes at 28 °C for 7 days. After carefully removing the liquid culture, the tubes were stained with 4 mL of 0.1% (*w*/*v*) crystal violet for 30 min. Unbound dye was removed by gently rinsing the tubes three times with sterile distilled water. The stained tubes were air-dried at 37 °C for 2 h. Biofilm formation was visually observed and photographed. For quantification, the bound crystal violet was solubilized with 1 mL of elution solution (40% methanol, 10% glacial acetic acid), and the absorbance of the resulting solution was measured at 575 nm. The *Pectobacterium carotovorum* PccS1 strain (provided from Nanjing agricultural university) was used as a positive control. Each assay was performed with nine replicates in total.

### 2.10. Cell Wall-Degrading Enzyme Activity Assay

The cell wall-degrading enzymes activity assay was performed as previously described [27]. Here, the *Pectobacterium carotovorum* PccS1 strain (provided by Nanjing agricultural university) was used as a positive control. The activity of cell wall-degrading enzymes was assessed based on the formation of clear halos around bacterial colonies spotted onto assay plates and incubated at 28 °C for 48 h. Subsequently, the plates were photographed, and the halo areas (cm^2^) were measured. Each assay was repeated with three to five replicates.

### 2.11. Genome Sequencing, Assembly, Annotation and Bioinformatics Analysis

Following the manufacturer’s instructions, we extracted genomic DNA with a commercial kit (Tiangen, Beijing, China). DNA quality and quantity were assessed using a NanoDrop spectrophotometer and a Qubit^®^ dsDNA HS Assay Kit (Thermo Fisher Scientific, Waltham, MA, USA).

Whole-genome sequencing was performed using a hybrid strategy combining Illumina paired-end and PacBio RS II single-molecule real-time (SMRT) platforms (Majorbio Bio-Pharm Technology Co., Ltd., Shanghai, China). The assembled genome was annotated through a multi-database approach. Protein-coding genes were functionally annotated by searching against the NCBI non-redundant (Nr), UniProt/Swiss-Prot, KEGG, GO, and COG databases. Protein family analysis was conducted using Pfam_Scan based on the Pfam database [32]. Additional functional annotations were obtained from the CAZy (Carbohydrate-Active enZYmes), PHI (Pathogen–Host Interactions), and CARD (Comprehensive Antibiotic Resistance Database) databases. Secondary metabolite biosynthetic gene clusters were identified with antiSMASH [33]. Two-component systems (TCSs) were predicted based on conserved domain structures [34]. Secretory and transmembrane proteins were predicted using SignalP 4.0 [35] and TMHMM [36], respectively.

### 2.12. Average Nucleotide Identity (ANI) Comparative Genomic Analysis

Average Nucleotide Identity (ANI) values were calculated on the website (http://enve-omics.ce.gatech.edu/ani/) (accessed on 26 September 2025). ANI values were computed and a phylogenetic heatmap was subsequently generated and analyzed using GraphPad Prism 9.5 (GraphPad Software, San Diego, CA, USA).

### 2.13. LC–MS Analysis of Fermentation Broth

A total of 18 samples were analyzed by liquid chromatography–tandem mass spectrometry (LC-MS/MS). The raw data files were processed using Progenesis QI software 2.0 (Waters Corporation, Milford, MA, USA) for peak detection, alignment, and integration. Metabolites were annotated by matching mass spectra against the HMDB and Metlin databases, as well as a custom in-house library.

To eliminate or minimize experimental and analytical variations, the annotated data underwent a series of preprocessing steps. First, features with a missing value rate >20% within any sample group were removed. Subsequently, the remaining missing values were imputed using the minimum value found across all samples. The response intensities of the mass spectral peaks were then normalized using the total sum normalization method, yielding a normalized data matrix. Furthermore, variables with a relative standard deviation (RSD) >30% in the quality control (QC) samples were filtered out. Finally, the data matrix was subjected to a log10 transformation, resulting in the final processed dataset used for all subsequent analyses.

Statistical analyses, including principal component analysis (PCA) and orthogonal partial least squares discriminant analysis (OPLS-DA), were performed using the ropls package (v1.6.2) in R. Functional annotation of metabolites was conducted by referencing the HMDB and KEGG databases.

### 2.14. Statistical Analysis

Statistical analyses are carried out by GraphPad software and assessed by paired T test, one-way ANOVA, and two-way ANOVA followed by Duncan’s or Šídák’s multiple comparisons post hoc test.

## 3. Results

### 3.1. Screening of Paenibacillus polymyxa JX-1

**Figure 1:**
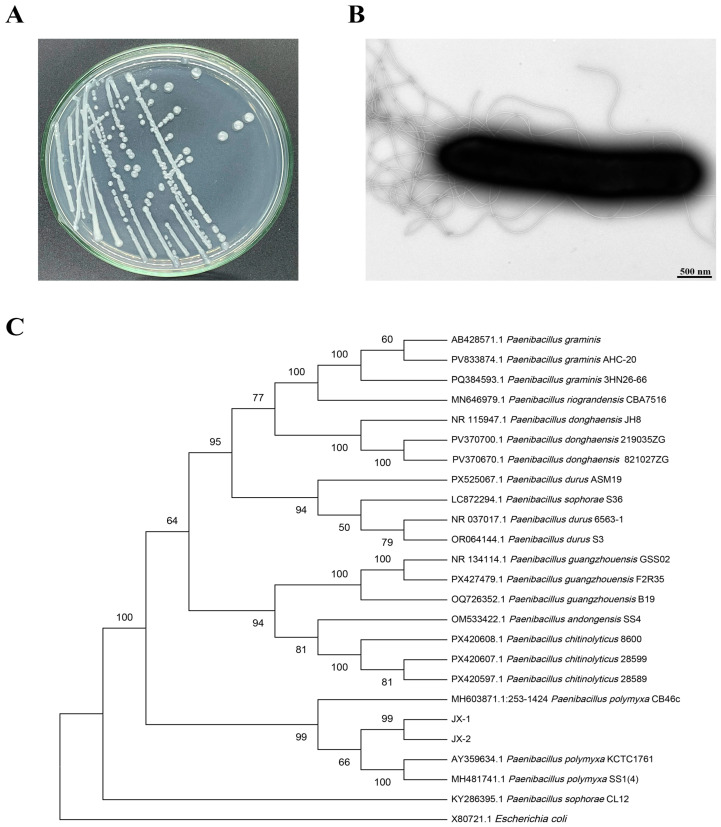
(**A**) Strain JX-1 colony on PDA medium; (**B**) morphological features of JX-1 by transmission electron microscopy (TEM); (**C**) neighbor-joining phylogenetic tree showing the phylogenetic relationship of strain JX-1 with other members of the *Paenibacillus* genus based on 16S rRNA gene sequences. The gene sequences of *E*. *coli* (X80721.1) were utilized as the root for constructing the phylogenetic tree. The bootstrap consensus was inferred from 1000 replicates.

The strain JX-1 was isolated from infected eggplant stems showing mild wilting in the field. The colony of strain JX-1 cultured on PDA medium displayed a circular morphology with an opaque white appearance (Figure 1A). Transmission electron microscopy (TEM) further revealed that the bacterial cells were rod-shaped and elliptical, featuring peritrichous flagella (Figure 1B). Furthermore, phylogenetic analysis based on 16S rRNA gene sequencing implied that this strain belonged to *P. polymyxa* (Figure 1C).

### 3.2. Paenibacillus polymyxa JX-1 Significantly Reduces Clubroot Disease in Both Arabidopsis thaliana and Brassica rapa in Planta

**Figure 2:**
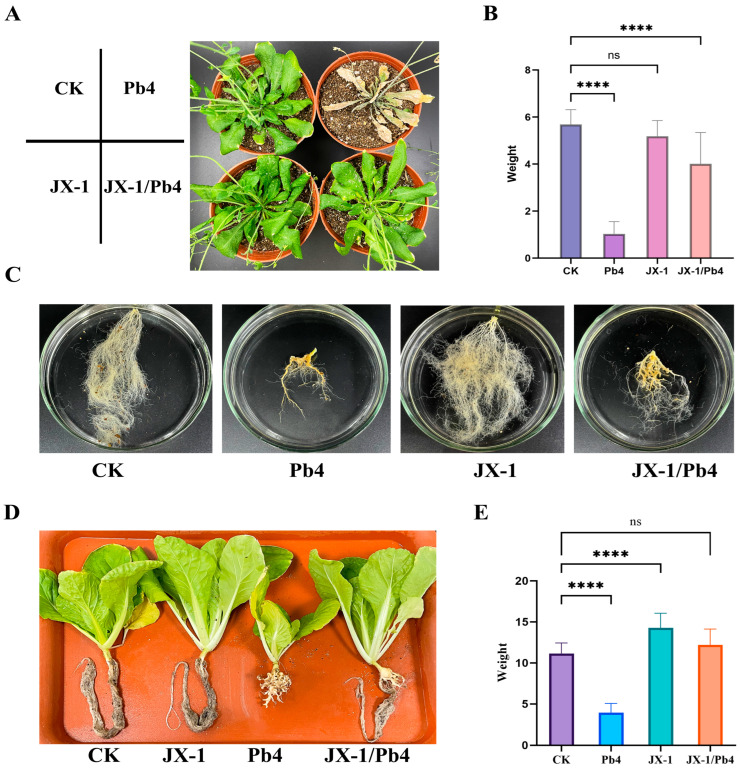
Control of clubroot disease by *Paenibacillus polymixa* JX-1 in both *Arabidopsis thaliana* and *Brassica rapa* in planta. The image displays *A. thaliana* subjected to various treatments, including the application of sterile culture medium as a control, the application of JX-1 bacterial solution alone, the inoculation with *Plasmodiophora brassicae* Pb4 spore suspension alone, and the application of JX-1 followed by inoculation with Pb4 spore suspension. (**B**) Analysis of fresh weight of *A. thaliana* corresponding to different treatments in (**A**). **** *p* value < 0.0001; (**C**) Images of *A. thaliana* roots corresponding to (**A**). (**D**) The image displays *B. rapa* subjected to various treatments, including the application of sterile culture medium as a control, the application of JX-1 bacterial solution alone, the inoculation with Pb4 spore suspension alone and the application of JX-1 followed by inoculation with Pb4 spore suspension. (**E**) Analysis of fresh weight data of *B. rapa* corresponding to different treatments in *D. Values* are mean ± SD (*n* = 6). **** *p* value < 0.0001. ns indicates no significant difference.

To explore the antagonistic range of strain JX-1, we conducted experiments on *Plasmodiophora brassicae*, a protist biotrophic pathogen that cannot be cultured in vitro. Given the biotrophic nature of *P. brassicae*, we performed in planta antagonistic experiments by directly inoculating the plants with the pathogen and strain JX-1. At four weeks post-inoculation, JX-1 treatment markedly alleviated disease symptoms. Compared to the stunted growth and high mortality observed in plants infected with *P. brassicae* Pb4 alone, JX-1-treated plants maintained healthy growth (Figure 2). This protective effect was associated with a significant promotion of root development, particularly in fibrous root growth, and a restoration of shoot fresh weight (Figure 2B,C). The consistent results from both *A. thaliana* and *B. rapa* confirmed the broad efficacy of JX-1 across different host plants (Figure 2D,E).

### 3.3. In Planta Biocontrol Assay Demonstrates the Preventive Efficacy of JX-1 Fermentation Filtrate Against Clubroot Disease

The biocontrol efficacy of *P. polymyxa* JX-1 against clubroot was further validated in planta using its cell-free fermentation filtrate (7-day YPDA culture). Four treatment groups were established: (1) YPDA medium control (CK), (2) pathogen inoculation with *P. brassicae* spore suspension (Pb4), (3) pre-treatment with JX-1 filtrate followed by pathogen inoculation (Pb4 + JX-1), and (4) treatment with JX-1 filtrate alone (JX-1).

**Figure 3:**
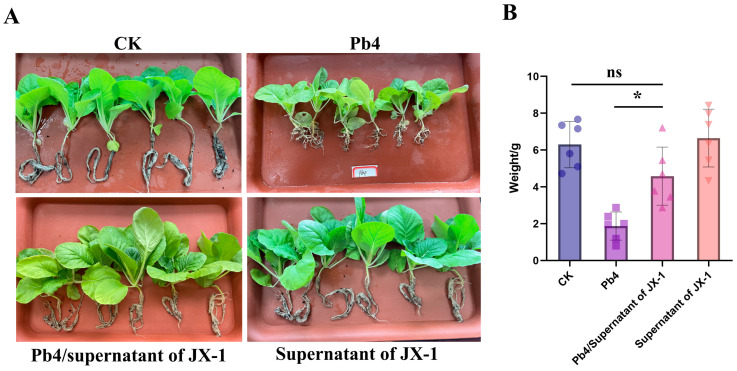
In planta biocontrol efficacy of the fermentation filtrate from *Paenibacillus polymyxa* JX-1 against clubroot disease. (**A**) Representative phenotypes of plant roots subjected to different treatments: CK (water control), Pb4 (inoculation with Plasmodiophora brassicae spore suspension), Pb4 + JX-1 (pre-treatment with JX-1 fermentation filtrate followed by pathogen inoculation), and JX-1 (treatment with JX-1 filtrate alone). The pre-treatment with JX-1 filtrate prevented the formation of root galls. (**B**) Fresh weight of plants from the four treatment groups. Values are mean ± SD (*n* = 6). * *p* < 0.05. ns indicates no significant difference.

Plant phenotypic assessment revealed a significant difference among treatments (Figure 3). Plants in the Pb4 group developed severe clubroot symptoms, including pronounced root galling. In contrast, pre-treatment with JX-1 filtrate conferred significant protection, with root systems remaining healthy and largely symptom-free, comparable to both CK and the JX-1 filtrate-only group (Figure 3A). The absence of symptoms in the latter two groups confirmed that the filtrate itself was non-phytotoxic.

Consistent with the phenotypic observations, the fresh weight biomass of plants was significantly affected by the treatments (Figure 3B). Pathogen inoculation (Pb4) alone caused a significant reduction in plant fresh weight compared to the control (Figure 3B). However, pre-application of the JX-1 fermentation filtrate (Pb4 + JX-1) effectively prevented this biomass loss, restoring plant fresh weight to a level statistically equivalent to that of the healthy control (CK) and the filtrate-only (JX-1) group (Figure 3B). This result confirms that the antimicrobial metabolites present in the JX-1 fermentation filtrate can systemically protect host plants from *P. brassicae* infection and the associated growth suppression.

### 3.4. Paenibacillus polymyxa JX-1 and Its Metabolic Products Effectively Inhibit the Growth of Various Plant Pathogens

To comprehensively assess the biocontrol potential of *P. polymyxa* JX-1, its antagonistic activity was evaluated in vitro using three distinct experimental approaches.

**Figure 4:**
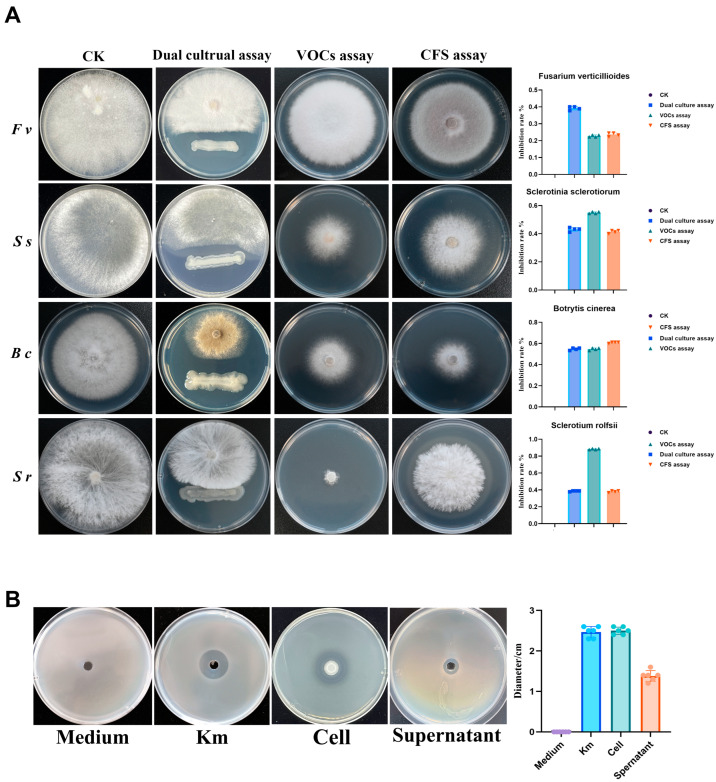
In vitro antagonistic activity of *Paenibacillus polymyxa* JX-1 against soil-borne fungal and bacterial pathogens. (**A**) Antagonism assays of strain JX-1 against four common soil-borne fungi: *Fusarium verticillioides*, *Sclerotinia sclerotiorum*, *Botrytis cinerea*, and *Sclerotium rolfsii*. For each fungus, four treatments are presented from left to right: control (CK), dual-culture confrontation, volatile organic compound (VOC) inhibition, and cell-free supernatant (CFS) activity. The rightmost panel shows the corresponding quantitative inhibition rates (%) for each treatment. Values are mean ± SD (*n* = 4). (**B**) Antibacterial activity of strain JX-1 against the soil-borne bacterial pathogen *Pectobacterium* PccS1 in a well-diffusion assay. From left to right: control (CK, sterile medium), positive control with kanamycin (Km), inhibition zone produced by spotting 2 μL of JX-1 cell suspension, and inhibition zone resulting from adding 10 μL of JX-1 CFS into a well. The right panel displays the diameter of inhibition zones (mm). Values are mean ± SD (*n* = 6).

In dual-culture plate assays, strain JX-1 demonstrated significant antagonism against four common soil-borne phytopathogenic fungi, with mycelial growth inhibition rates of 39.2 ± 1.1% for *Fusarium verticillioides*, 43.1 ± 1.4% for *Sclerotinia sclerotiorum*, 54.7 ± 1.1% for *Botrytis cinerea*, and 38.7 ± 0.5% for *Sclerotium rolfsii* (Figure 4A, second column).

In a partitioned plate assay, the volatile organic compounds (VOCs) emitted by JX-1 also effectively inhibited the mycelial growth of all tested fungi. The inhibition was particularly strong against *Sclerotinia sclerotiorum* 55.0 ± 0.6%, *Botrytis cinerea* 54.7 ± 1.6%, and *Sclerotium rolfsii* 88.3 ± 0.06%, confirming a potent non-contact antagonistic mechanism (Figure 4A, third column).

Furthermore, the cell-free culture supernatant (CFS) of JX-1 displayed potent and differential antifungal activity. It was most effective against *Sclerotinia sclerotiorum* and *Botrytis cinerea*, achieving inhibition rates of 41.4 ± 1.0% and 60.8 ± 0.5%, respectively, thereby validating the presence of potent soluble inhibitory metabolites (Figure 4A, fourth column). The inhibition rates for all three antagonistic modes were quantitatively compared to the control for each pathogen (Figure 4A, right panel).

The antagonistic activity of JX-1 extended to bacterial pathogens. In a well-diffusion assay, both a cell suspension and CFS produced distinct inhibition zones against the soft rot pathogen *Pectobacterium* PccS1, with a kanamycin as a positive control (Figure 4B). The inhibition zones diameters were significantly larger than those of the sterile medium control (Figure 4B, right panel).

Collectively, these results demonstrate that *P. polymyxa* JX-1 employs a multifaceted strategy encompassing direct interaction, volatile emission, and secretion of soluble compounds to exert broad-spectrum and, in some cases, pathogen-specific inhibitory effects against diverse fungal and bacterial pathogens.

### 3.5. Protease Production Correlates with the Biocontrol Mechanism of Paenibacillus polymyxa JX-1 Against Clubroot Disease

**Figure 5:**
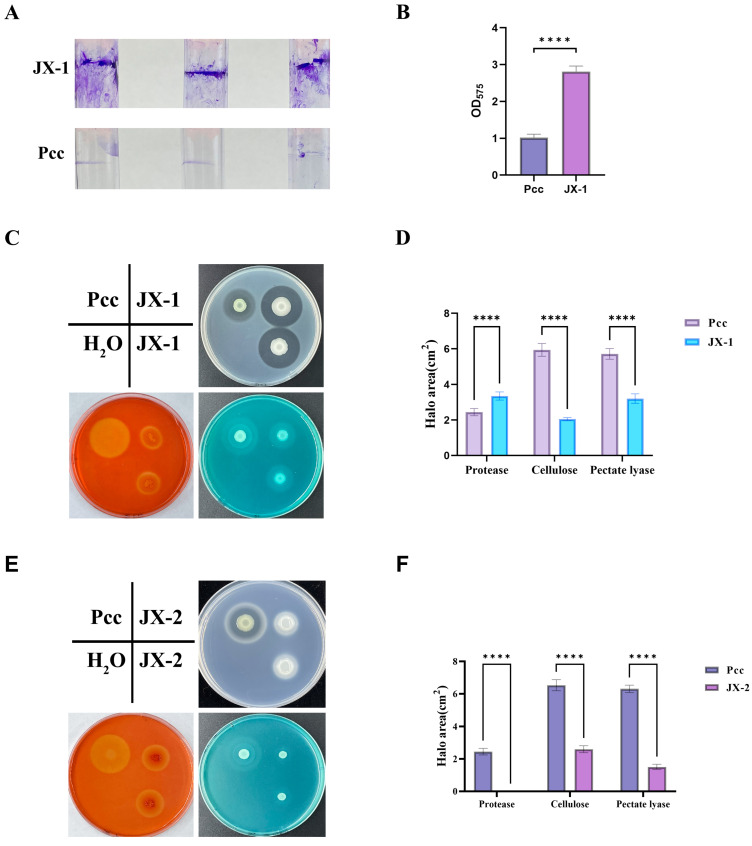
Bacterial extracellular enzymes and biofilm assay of strain JX-1. (**A**) Biofilm assay; (**B**) statistical analysis of absorbance detection after crystal violet elution of biofilm. Values are mean ± SD (*n* = 9). **** p < 0.0001; (**C**) bacterial extracellular enzymes of JX-1 including proteases (the white medium), cellulases (the red medium), and pectinases (the blue medium) assay; (**D**) statistical analysis of the results shown in (**C**); **** p < 0.0001; (**E**) bacterial extracellular enzymes of JX-2 including proteases, cellulases, and pectinases assay; (**F**) statistical analysis of the results showed in (**E**). Values are mean ± SD (*n* = 3). **** p < 0.0001.

Not all *P. polymyxa* exhibit biocontrol efficacy against clubroot disease. For example, strain JX-2, isolated from the same plant as JX-1, showed no antagonism toward *P. brassicae*. To investigate the functional components associated to disease suppression, we assayed extracellular enzyme activities (protease, cellulase, and pectinase) and biofilm formation in strain JX-1 (Figure 5A–D).

The biofilm formation result indicated that JX-1 has a greater capacity to effectively colonize and persist on plant root surfaces (Figure 5A,B). The assays with the pathogen causing soft rot, *Pectobacterium carotovorum* PccS1, as a positive control, demonstrated that JX-1 produced protease, cellulase, and pectinase, with protease activity exceeding that of the soft rot pathogen (Figure 5C,D).

Comparative analysis with a non-antagonistic strain named *P. polymyxa* JX-2, which lacked efficacy against *P. brassicae*, revealed that JX-2 retained cellulase and pectinase production but failed to secrete protease (Figure 5E,F). Furthermore, the supernatant of JX-2 did not inhibit the growth of PccS1 (Figure S1).

These results may suggest that the proteases or other metabolites present in the supernatant of JX-1 may be associated with its ability to reduce the severity of clubroot disease. However, future work involving mutagenesis of the protease genes is required to establish a definitive causal relationship.

### 3.6. Genome Sequencing of Paenibacillus polymyxa JX-1

**Figure 6:**
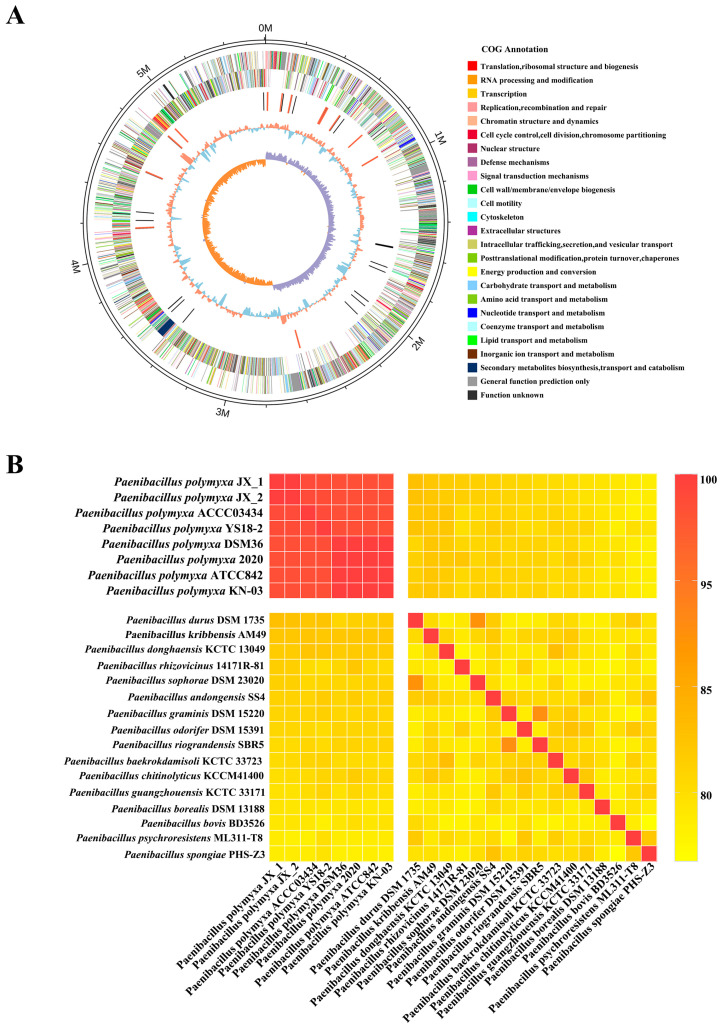
Genome map and species confirmation of JX-1. (**A**) Genome map of strain JX-1: the outermost circle represents the genomic sequence position coordinates. The outer to inner circles represent genes on the positive strand, genes on the negative strand, ncRNA (black for tRNA, red for rRNA), GC content (red for values above the mean, blue for values below the mean), and GC skew (used to measure the relative content of G and C and to mark the origin and terminus in circular chromosomes; GC skew = (G − C)/(G + C); purple for values greater than 0, orange for values less than 0). (**B**) A heatmap was created to represent the pairwise Average Nucleotide Identity (ANI) values between the complete genomes of *P. polymyxa* JX-1 and a comparative group of 23 other *Paenibacillus* species including *P. polymyxa* JX-2, offering a visual depiction of the genetic relatedness among these bacterial strains.

To achieve a comprehensive understanding of the phylogeny and function of the *P. polymyxa* JX-1, we sequenced and assembled its complete genome (Figure 6A). We sequenced and assembled the complete genome of strain JX-1 to elucidate its phylogenetic and functional potential (Figure 6A). Using a hybrid sequencing strategy combining Illumina paired-end and PacBio RS II SMRT technologies, we generated a total of 1,555,444,452 high-quality reads (Table 2). The final assembly yielded a single, circular chromosome of 5,605,467 bp with a GC content of 46.50% and contained no gaps. A total of 4845 protein-coding genes were predicted, accounting for 85.31% of the genome. Additionally, we identified 39 rRNA, 109 tRNA, and 3 sRNA genes, along with 12 genomic islands and 1 prophage (Table 2). Functional annotation assigned 4820, 3111, 2570, 3875, 3956, and 4042 coding sequences to the Nr, Swiss-Prot, KEGG, COG, GO, and Pfam databases, respectively (Figure S2, Table 2).

### 3.7. Average Nucleotide Identity (ANI) Analysis Revealed That Strain JX-1 Is a Member of Paenibacillus polymyxa

ANI is a robust method for assessing evolutionary distances among bacteria. In this study, we applied this method to identify JX-1 at the species level. The ANI value between *P. polymyxa* JX-1 and the reference strain *P. polymyxa* ATCC 842 was found to be 98.36% (Figure 6B). Furthermore, JX-1 exhibited ANI values above the 95% species threshold not only with JX-2 (99.96%) but also with six other conspecific *P. polymyxa* strains (Figure 6B). Conversely, ANI values between JX-1 and sixteen other type strains from various *Paenibacillus* species were all below 90% (Figure 6B), clearly distinguishing it from other species within the genus. These results collectively confirm that strain JX-1 belongs to the species *Paenibacillus polymyxa*. The reference strains used for the ANI analysis, along with their names and NCBI accession numbers, are presented in Table 3.

### 3.8. Analysis of Carbohydrate-Active Enzymes (CAZyme), Proteases and Secondary Metabolites Genes in the Genome of Paenibacillus polymyxa JX-1

**Figure 7:**
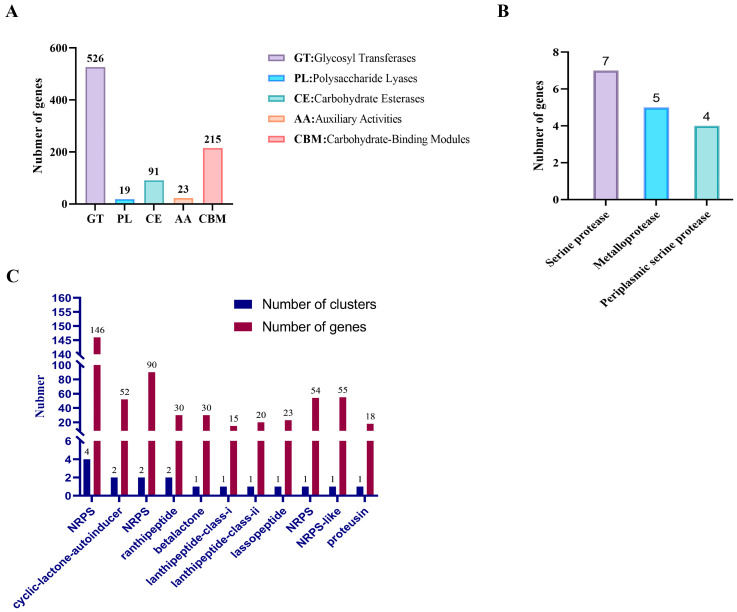
(**A**) Functional characterization of the glycoside hydrolase family is based on the CAZy database. (**B**) The predicted numbers of three types of extracellular hydrolytic proteases of JX-1. (**C**) The hypothetical gene cluster lists encoding the secondary metabolites predicted by antiSMASH in the genomes of JX-1.

Genomic analysis of strain JX-1 identified 686 putative carbohydrate-active enzymes (CAZymes), primarily consisting of 647 glycoside hydrolases (GHs) and 526 glycosyl transferases (GTs) (Figure 7A).

Meanwhile, we analyzed the protease genes potentially involved in protein degradation and found a total of 16 protein genes that may participate in this function: 7 Serine proteases, 5 Metalloproteases, and 4 Periplasmic serine proteases (Figure 7B). This strongly corroborates the previous experimental results: JX-1 can secrete a large amount of extracellular proteases to degrade casein in milk (Figure 5C,D).

Additionally, genomic prediction revealed 17 biosynthetic gene clusters (BGCs) encoding secondary metabolites with putative antibiotic activities (Figure 7C). These comprise 6 non-ribosomal peptide synthetase (NRPS) clusters, 2 trans-AT polyketide synthase (transAT-PKS) clusters, 2 cyclic lactone autoinducer clusters, 2 lanthipeptide clusters (Class I and II), individual clusters for ranthipeptide, betalactone, lassopeptide, NRPS-PKS hybrid, type III PKS (T3PKS), NRPS-like cyclic lactone autoinducer, and proteusin. NRPS pathways contained the highest number of clusters and associated genes, while other compound classes were represented by one or two BGCs.

### 3.9. Comparative Metabolomics Identifies a Specialized Antimicrobial Metabolome in the Broad-Spectrum Biocontrol Strain P. polymyxa JX-1

**Figure 8:**
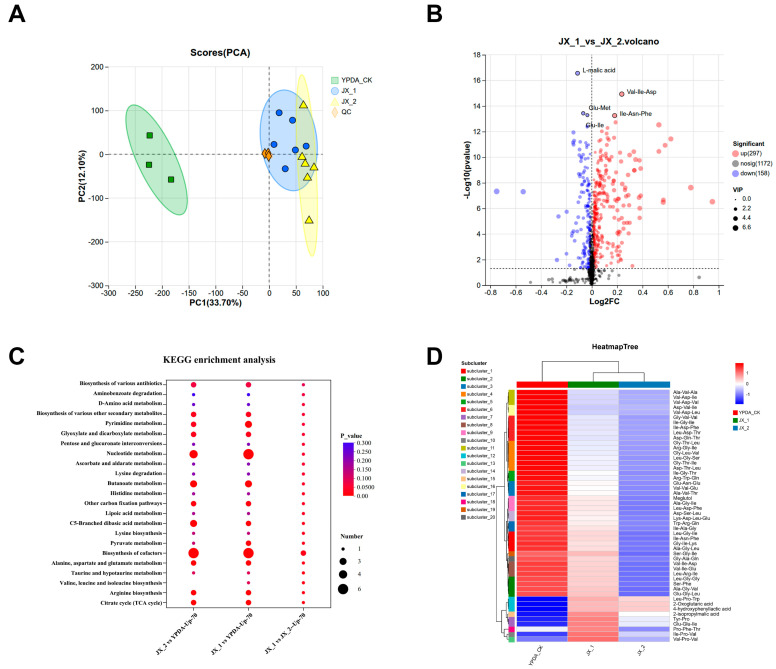
Comparative metabolomic analysis of *Paenibacillus polymyxa* strains JX-1, JX-2 and YPDA. (**A**) Principal component analysis (PCA) score plot of metabolic profiles derived from the fermentation broths of strains JX-1 and JX-2. Each point represents an independent biological replicate. (**B**) Volcano plot depicting differentially accumulated metabolites between JX-1 and JX-2. Each point represents a metabolite. Red points indicate significantly upregulated metabolites in JX-1 (fold change > [threshold] and *p*-value < 0.05), blue points indicate significantly downregulated metabolites, and gray points represent non-significant changes. The dashed lines mark the thresholds for statistical significance and fold change. (**C**) KEGG pathway enrichment analysis of metabolites upregulated in strains JX-1 and JX-2 relative to a YPDA medium control. The top enriched pathways are shown. The color scale represents the −log10 (*p*-value), and the dot size corresponds to the enrichment ratio. (**D**) Hierarchical clustering heatmap of metabolites significantly upregulated in JX-1 compared to JX-2 after stringent filtering (confident database match and MS/MS score ≥ 70). The color scale from blue to red indicates relative abundance from low to high across samples (JX-1, YPDA and JX-2 replicates). Key annotated antimicrobial compounds or peptide classes are indicated on the right.

To identify the metabolic foundation of the distinct biocontrol phenotypes between strains, we performed a comparative metabolomic analysis on *P. polymyxa* JX-1 (broad-spectrum antagonist with clubroot disease activity) and its conspecific strain JX-2 (lacking clubroot disease suppression). Principal component analysis (PCA) of their extracellular metabolomes revealed a clear and significant separation between JX-1 and JX-2 along the first principal component, indicating fundamental differences in their metabolic outputs (Figure 8A). A global volcano plot analysis further quantified this disparity, showing a markedly greater number of metabolites upregulated in JX-1 compared to JX-2, highlighting its more active metabolic state (Figure 8B).

A stringent two-step filter was applied to ensure high-confidence identifications: (i) matching to reference mass spectral databases, and (ii) an MS/MS fragmentation score ≥ 70. The resulting high-confidence dataset was used for all subsequent analyses.

To understand the functional orientation of the filtered metabolite profiles, KEGG pathway enrichment analysis was performed on metabolites upregulated in each strain relative to the sterile YPDA medium control. The analysis demonstrated that JX-1 specifically enriched pathways directly linked to antimicrobial production and defense, such as Pyrimidine metabolism, Nucleotide metabolism, valine leucine isoleucine biosynthesis and Pyruvate metabolism (Figure 8C). In contrast, pathways enriched in JX-2 were less directly associated with antagonism. This suggests that JX-1’s metabolism is intrinsically reprogrammed toward generating a chemical arsenal for biocontrol.

Hierarchical clustering of the high-confidence metabolites significantly upregulated in JX-1 (versus both the YPDA control and the conspecific strain JX-2) revealed distinct metabolite clusters that were highly abundant in JX-1 but present at low or undetectable levels in the control and JX-2 (Figure 8D). These JX-1-specific clusters were notably enriched with small peptides (Leu-Pro-Trp, Tyr-Pro, Glu-Glu-Ile, Pro-Phe-Thr, Ile-Pro-Val, and Val-Pro-Val) characterized by a high proportion of hydrophobic amino acids—a structural feature commonly associated with membrane interaction and potential antimicrobial activity. Additionally, these clusters contained putative antimicrobial organic acids, including 2-Oxoglutaric acid, 4-hydroxyphenyllactic acid, and 2-isopropylmalic acid, which are often implicated in microbial antagonism.

Together, this metabolomic profile delineates a unique chemical arsenal in JX-1, comprising both hydrophobic peptides and antimicrobial organic acids, which collectively provide a molecular basis for its superior and broad-spectrum biocontrol capability, including its pronounced activity against *P. brassicae*. The direct antimicrobial function of these identified peptides, however, requires further experimental validation.

## 4. Discussion

*Paenibacillus polymyxa* has been extensively documented in the biocontrol literature for suppressing diverse fungal pathogens [16,26,37,38,39,40,41]. Although prior studies have indicated the potential of *P. polymyxa* in managing clubroot disease, a significant knowledge gap remains [5,42]. No *P. polymyxa* strain has been comprehensively documented to possess concurrent, broad-spectrum efficacy against diverse soil-borne pathogens (bacterial, fungal, and protist). The specific mechanisms underlying its activity against the obligate parasite *Plasmodiophora brassicae* are particularly obscure. This study addresses these gaps by characterizing the novel strain JX-1 through an integrated multi-omics and comparative phenotypic approach, revealing a multifaceted antagonistic strategy and proposing a key mechanistic determinant for clubroot suppression.

Our findings establish *P. polymyxa* JX-1 as a potent, broad-spectrum antagonist. The initial isolation from mildly symptomatic plants in the field suggested its potential as an adapted, indigenous antagonist. Subsequent in planta experiments confirmed that JX-1 significantly alleviated clubroot severity and restored plant biomass (Figure 2). Notably, its cell-free supernatant alone also sufficiently suppress disease, indicating the presence of potent soluble antagonistic compounds (Figure 3). Consistent with the established biocontrol profile of this species [26,40,43], JX-1 exhibited strong in vitro inhibition against a range of phytopathogenic fungi, including *Fusarium verticillioides*, *Sclerotinia sclerotiorum*, *Botrytis cinerea*, and *Sclerotium rolfsii* (Figure 4). Notably, JX-1’s antagonistic capacity extends beyond diffusible metabolites to include volatile organic compounds (VOCs), which significantly inhibited the hyphal growth of *S. rolfsii* (Figure 4A, inhibition rate 88%). We report for the first time the antagonistic activity of a *Paenibacillus polymyxa* strain (JX-1) against *S. rolfsii*, expanding the known biocontrol spectrum of this species beyond previously reported pathogens [44,45,46].

Its cell-free supernatant also showed potent activity, achieving 61% inhibition against *B. cinerea* (Figure 4A). Furthermore, JX-1 secreted a large amount of cell wall-degrading enzymes including proteases, cellulases, and pectinases (Figure 5). Importantly, protease secretion was markedly higher in JX-1 than in the non-antagonistic strain JX-2. Proteases are known to exhibit insecticidal activity against insects [47], and many plant growth-promoting biocontrol bacterial strains can secrete proteases [48]. All these results suggest that JX-1 has the ability to secrete a large amount of biocontrol substances to achieve antagonistic effects.

Whole-genome sequencing provided the genetic blueprint for this functional capacity (Figure 6A). Average Nucleotide Identity (ANI) analysis definitively identified JX-1 as *P. polymyxa* (Figure 6B). Genome mining revealed a rich arsenal of biocontrol-associated genes, including multiple biosynthetic gene clusters (BGCs) for non-ribosomal peptide synthesis (NRPS) and a substantial complement of CAZymes (Figure 7). These findings genetically underpinned its potential to produce diverse antimicrobial metabolites.

Despite high genomic similarity with JX-2 (ANI = 99.6%), JX-1 appears to have undergone adaptive variations, potentially enabling enhanced production of proteases and other secondary metabolites for pathogen defense. This divergence underscores the utility of comparative omics for pinpointing antimicrobial determinants.

Therefore, we conducted a comparative non-targeted metabolomic analysis of the JX-1 supernatant. Using the closely related, clubroot-inactive strain JX-2—isolated from the same host—alongside a YPDA medium control, we filtered out background and conserved metabolites, thereby pinpointing those uniquely associated with the biocontrol phenotype. The results revealed a significant accumulation of hydrophobic small peptides and phenolic acid derivatives specifically in the JX-1. These compounds are likely products of the annotated NRPS pathways.

The KEGG enrichment analysis of differentially accumulated metabolites in strain JX-1 reveals a targeted upregulation of several core metabolic pathways, including Pyrimidine metabolism, Nucleotide metabolism, valine leucine isoleucine biosynthesis and Pyruvate metabolism (Figure 8C). This coordinated metabolic reprogramming provides a functional context for the observed antagonistic phenotype and aligns with the genomic and metabolomic findings. The upregulation of valine, leucine, and isoleucine biosynthesis is of particular significance in JX-1. These branched-chain amino acids serve as direct precursors for protein synthesis and, notably, are key hydrophobic components of antimicrobial peptides [49]. For example, *Bacillus velezensis* F85 and *Bacillus amyloliquefaciens* T113 revealed that they possess genes responsible for antibiotic biosynthesis and antimicrobial peptide production [18]. This finding offers a clear metabolic explanation for the earlier identification of hydrophobic-amino-acid-containing small peptides in the JX-1 supernatant, directly linking a specific biosynthetic pathway to the production of putative effector molecules that may disrupt pathogen membranes.

Concurrently, the enhanced nucleotide and pyrimidine metabolism likely supports the high transcriptional and translational demand required for the rapid synthesis of these antimicrobial peptides, cell wall-degrading enzymes, and other pathogen-responsive proteins.

Collectively, this metabolic profile suggests that JX-1 employs a strategic resource re-arrangement under antagonistic conditions. Carbon and nitrogen flux are redirected to prioritize the biosynthesis of amino acids and nucleotides. This building block is essential for manufacturing peptides and enzymes and maintaining cellular energy and redox balance. This integrated metabolic shift, from primary building block synthesis to potential antimicrobial compound output, underscores a sophisticated adaptation that differentiates JX-1 from its less effective JX-2. It thereby provides a systems-level understanding of JX-1’s superior biocontrol efficacy.

While this integrated approach strongly links specific metabolites to JX-1’s efficacy, future work is needed to validate their individual roles. The logical next steps include the purification and structural elucidation of the key peptides, proteases and phenolic compounds, followed by direct bioassay against *P. brassicae* and other pathogens. Finally, field trials are essential to evaluate the consistency and practicality of JX-1 application under complex soil conditions.

Taken together, these findings establish JX-1 as a potential biocontrol agent with three distinctive advantages: (i) its antimicrobial capacity to suppress diverse pathogens via both volatile and soluble metabolites; (ii) unprecedented broad-spectrum activity against fungi, bacteria, and protista pathogens; (iii) a proposed mechanism implicating extracellular proteases and small peptides in clubroot suppression, as supported by comparative phenotypic and multi-omics analyses. This correlation, while requiring further validation through mutagenesis studies, highlights a promising target for future mechanistic research. The identified protease functionality provides a tractable starting point for subsequent dissection of the mode of action and for potential strain improvement programs.

## 5. Conclusions

In this study, we isolated and characterized *Paenibacillus polymyxa* JX-1, a novel endophytic strain with potent, broad-spectrum biocontrol activity against diverse soil-borne fungi, bacteria, and the protist *Plasmodiophora brassicae*. Comparative phenotypic and multi-omics analyses, benchmarked against the closely related but inactive strain JX-2, highlighted heightened extracellular protease activity and the specific production of hydrophobic small peptides as key features associated with its unique efficacy. These traits, underpinned by a genomic repertoire rich in CAZymes and biosynthetic gene clusters and supported by a distinct metabolomic profile, suggest a multifaceted antagonistic strategy. Our findings point to a testable mechanistic hypothesis for clubroot suppression and position JX-1 as a promising candidate for the development of sustainable disease management solutions.

The primary limitation of this study is that the direct functional roles of the identified proteases and small peptides in antagonism, particularly against *P. brassicae*, remain to be experimentally confirmed. Therefore, future work should focus on (1) the purification and structural elucidation of the key proteases, peptide, and phenolic acid metabolites; (2) direct bioassays of these compounds against target pathogens; (3) mutagenesis studies to validate the role of specific protease and NRPS genes; and (4) field trials to evaluate the practical efficacy and consistency of JX-1 as a biocontrol inoculant under complex environmental conditions. This work not only provides a promising microbial resource but also delivers a clear, mechanism-focused roadmap for developing sustainable solutions against clubroot and other soil-borne diseases.

## Figures and Tables

**Table 1:** Source and origin of fungal pathogens used in this study.

Species Name	Strain Code	Host/Source	Location of Isolation	Date of Isolation
*Paenibacillus polymyxa*	JX-1	Mild rot eggplant stem	Ganzhou, China	December 2023
*Paenibacillus polymyxa*	JX-2	Mild rot eggplant stem	Ganzhou, China	December 2023
*Fusarium verticillioides*	D-2	Rot soybean root	Nanchang, China	August 2023
*Sclerotinia sclerotiorum*	QZ-1	Rot eggplant stem	Ganzhou, China	December 2023
*Botrytis cinerea*	SS-1	Rot mulberry	Nanchang, China	May 2024
*Sclerotium rolfsii*	JD-1	Rot hyacinth Bean root	Nanchang, China	July 2024
*Pectobacterium carotovorum*	PccS1	Nanjing agricultural university	Nanjing, China	2009

**Table 2:** Genome characteristics of strain JX-1.

Characteristics	Value
Genome size (bp)	5,605,467
GC content %	46.50
Chromosome	1
Number of contigs	1
Number of gaps	0
Mean read length (bp)	11,460
Gene-prediction number	4845
rRNA genes	39
tRNA genes	109
sRNA	3
Gene islands	12
Prophage	1
Nr	4820
Swiss-prot	3111
KEGG	2570
COG	3875
GO	3956
Pfam	4042

**Table 3:** Reference strains used for ANI analysis.

Species Name	Strain Code	Type Strain	Accession Number
*Paenibacillus polymyxa*	JX-1	No	Accession number will be released in 2026
*Paenibacillus polymyxa*	JX-2	No	Accession number will be released in 2026
*Paenibacillus polymyxa*	ACCC03434	No	GCF_030122785.1
*Paenibacillus polymyxa*	YS18-2	No	GCF_024584565.1
*Paenibacillus polymyxa*	DSM36	No	GCF_015710975.1
*Paenibacillus polymyxa*	2020	No	GCF_015710815.1
*Paenibacillus polymyxa*	ATCC842	Yes	GCF_022811565.1
*Paenibacillus polymyxa*	KN-03	No	GCF_029203955.1
*Paenibacillus durus*	DSM 1735	Yes	GCF_000756615.1
*Paenibacillus kribbensis*	AM49	Yes	GCF_002240415.1
*Paenibacillus donghaensis*	KCTC 13049	Yes	GCF_002192415.1
*Paenibacillus rhizovicinus*	14171R-81	Yes	GCF_010365285.1
*Paenibacillus sophorae*	DSM 23020	Yes	GCF_018966525.1
*Paenibacillus andongensis*	SS4	Yes	GCF_025369935.1
*Paenibacillus graminis*	DSM 15220	Yes	GCF_000758705.1
*Paenibacillus odorifer*	DSM 15391	Yes	GCF_000758725.1
*Paenibacillus riograndensis*	SBR5	Yes	GCF_000981585.1
*Paenibacillus baekrokdamisoli*	KCTC 33723	Yes	GCF_003945345.1
*Paenibacillus chitinolyticus*	KCCM41400	Yes	GCF_004117095.1
*Paenibacillus guangzhouensis*	KCTC 33171	Yes	GCF_009363075.1
*Paenibacillus borealis*	DSM 13188	Yes	GCF_000758665.1
*Paenibacillus bovis*	BD3526	Yes	GCF_001421015.1
*Paenibacillus psychroresistens*	ML311-T8	Yes	GCF_009728935.1
*Paenibacillus spongiae*	PHS-Z3	Yes	GCF_024734895.1

## Data Availability

Sequence data from this article can be found in GenBank at https://www.ncbi.nlm.nih.gov/datasets/genome/ (accessed on 22 May 2027, accession number CP190748). All other relevant data are within the paper.

## Supplementary Materials

The following supporting information can be downloaded at https://www.mdpi.com/article/10.3390/microorganisms14030520/s1, Figure S1. Antibacterial activity assay of supernatant of strain JX-2 against the soil-borne bacterial pathogen *Pectobacterium* PccS1 in a well-diffusion assay. From left to right: control (CK, sterile medium), positive control with kanamycin (Km) and inhibition zone resulting from adding 10 μL of JX-2 cell-free supernatant into a well. Figure S2. Basic annotation of JX-1. (A) Analysis and statistical plot of shared and unique annotations in the database. (B) GOC function classification. (C) GO function classification. (D) Top 20 species distribution plot in NR library.

## Abbreviations

The following abbreviations are used in this manuscript:

LB	Lysogeny Broth
LA	Luria Agar
PDA	Potato Dextrose Agar
VOCs	Volatile organic compounds
ANI	Average Nucleotide Identity
BCAs	Biological control agents
