# Supplementary figures and images for: Multi-Omics and Phenotypic Analysis Reveal Paenibacillus polymyxa JX-1 as a Broad-Spectrum Biocontrol Agent Against Clubroot Disease
Source: Microorganisms. 2026 Feb 24;14(3):520. doi: 10.3390/microorganisms14030520 (PMC13028954; doi:10.3390/microorganisms14030520)

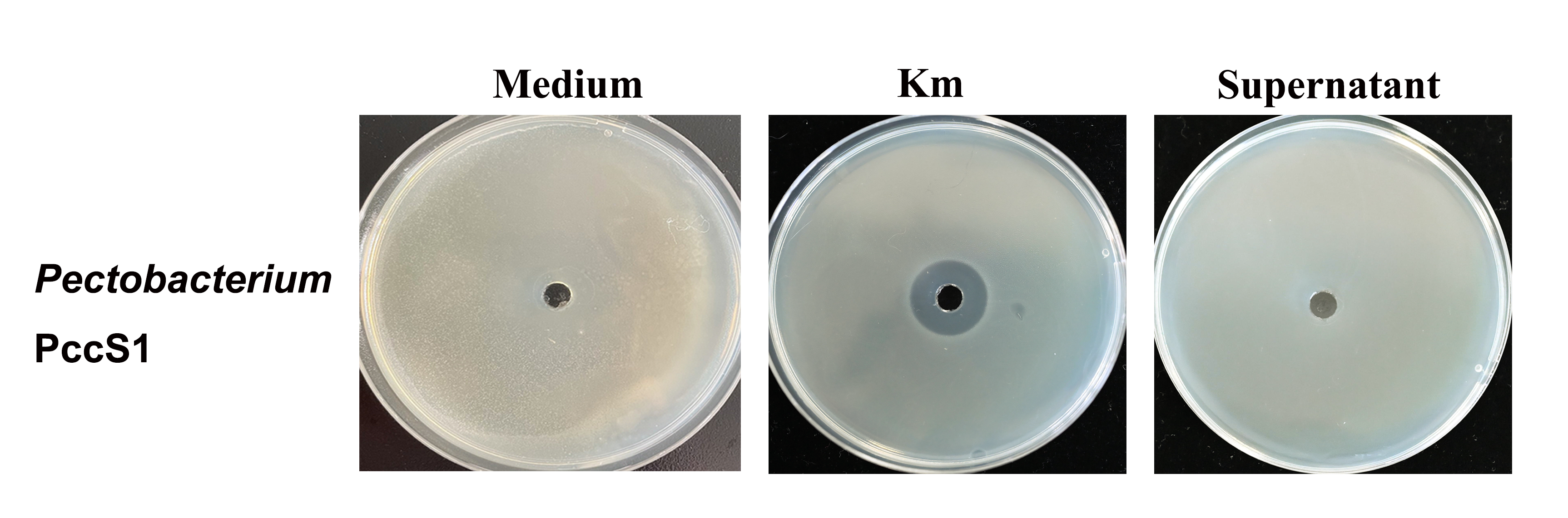

Supplement: Supplementary file 1 [file microorganisms-14-00520-s001.zip › Figure S1-2/Figure S1.tif]

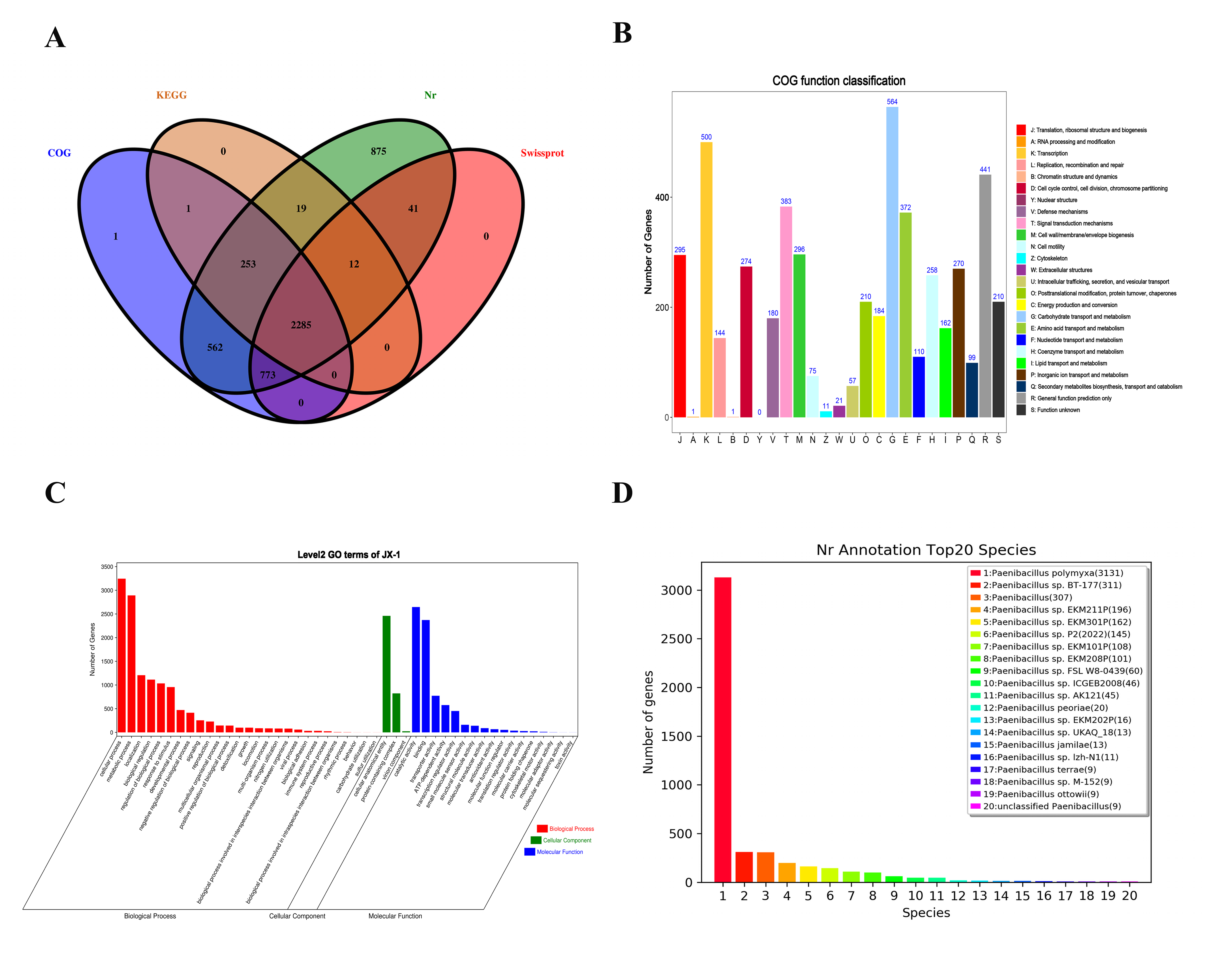

Supplement: Supplementary file 1 [file microorganisms-14-00520-s001.zip › Figure S1-2/Figure S2.tif]
